# 从结构到功能：凝血因子Ⅴ与组织因子途径抑制物α相互作用的新进展

**DOI:** 10.3760/cma.j.cn121090-20251112-00523

**Published:** 2026-08

**Authors:** 冰弦 李, 林花 杨, 刚 王

**Affiliations:** 山西医科大学第二临床医学院（第二医院）血液科，山西医科大学出凝血疾病及恶性血液病研究中心，血液病分子诊疗山西省重点实验室，太原 030001 Department of Hematology, the Second Hospital of Shanxi Medical University, Center for Shanxi Medical University and Tumor of the Hematopoietic and Lymphoid Tissues Diseases, Shanxi Provincial Key Laboratory for Molecular Diagnosis and Treatment of Hematological Diseases, Taiyuan 030001, China

## Abstract

凝血因子Ⅴ（FⅤ）与组织因子途径抑制物α（TFPIα）的相互作用是凝血系统促凝与抗凝平衡的关键节点。既往研究提示，FⅤB结构域的酸性区（AR2）与其碱性区（BR）或TFPIα C末端碱性尾结合是调控其功能状态的核心，但缺乏直接结构证据。近年来，冷冻电子显微镜技术首次解析了FⅤ-short及全长FⅤ的高分辨率结构，证实AR2表面形成负电性疏水平台，为BR或TFPIα结合提供分子基础。这些发现不仅验证了BR-AR2分子内相互作用在维持FⅤ未活化状态中的作用，也解释了FⅤ相关罕见疾病的病理机制，并使靶向FⅤ-TFPIα界面成为治疗出血性和血栓性疾病的新方向。

## Abstract (en)

The interaction between coagulation factor Ⅴ（FⅤ）and tissue factor pathway inhibitor alpha（TFPIα）is a central regulator of hemostatic balance. The acidic region（AR2）of the FⅤ B domain interacts with either its basic region（BR）or the basic C-terminal tail of TFPIα, modulating FⅤ activity. Recent cryo-electron microscopy studies have resolved the high-resolution structures of FⅤ-short and full-length FⅤ, revealing that AR2 forms a negatively charged hydrophobic platform that mediates BR or TFPIα binding. These insights confirm the BR-AR2 interaction as crucial for maintaining FⅤ in its inactive state and clarify the molecular basis of FⅤ-related disorders, highlighting the FⅤ-TFPIα interface as a promising therapeutic target for bleeding and thrombotic diseases.

凝血系统是维持止血与血流平衡的重要生理屏障，其核心内容是促凝与抗凝的精确调节[1]。在这一复杂的系统中，凝血因子Ⅴ（coagulation factor Ⅴ，FⅤ）起着独特的双重功效：既是促凝辅因子的前体[2]，又是组织因子途径抑制物α（tissue factor pathway inhibitor alpha，TFPIα）的主要结合蛋白和调节因子。FⅤ的关键调控中心位于B结构域，其中碱性区（basic region，BR）与酸性区（acidic region 2，AR2）借助分子内静电相互作用让其处于非活化状态[3]–[5]，而TFPIα通过自身C末端与AR2结合进而参与调控[6]。东德克萨斯出血性疾病（east Texas bleeding disorder）等遗传病例直接佐证了该相互作用的生理重要性[7]–[8]。由于B结构域存在高度无序现象，其具体分子机制长期缺乏结构学方面的证据，但近年来冷冻电子显微镜（电镜）技术的突破使解析这一难题有了全新的视角[9]–[10]。本文综述FⅤ与TFPIα的分子结构、功能特征及其相互作用的研究进展，探讨最新结构学发现对凝血调控和相关疾病机制的启示。

一、FⅤ

FⅤ由位于1号染色体长臂（1q24.2）的F5基因编码，主要在肝脏合成后释放入血浆中，是凝血级联反应中的关键辅助成分。前体FⅤ由2 196个氨基酸组成，相对分子质量约330×10^3^，结构域布局为A1-A2-B-A3-C1-C2，与FⅧ高度同源[11]–[13]。其中A1-A2构成重链，A3-C1-C2构成轻链，B结构域富含糖基，负责维持FⅤ的非活化状态[14]–[15]。凝血酶在R709、R1018和R1545位点依次切割，B结构域被移除，单链前体转化为由重链与轻链经Ca²⁺依赖性相互作用形成的活化异二聚体FⅤa[16]–[17]。FⅤa作为促凝辅因子，与FⅩa、磷脂膜及Ca²⁺共同组装凝血酶原酶复合物，显著提升凝血酶原激活速率，达成凝血反应信号放大[18]–[20]。除促凝作用外，FⅤ亦参与抗凝调节：FⅤa可作为活化蛋白C（activated protein C，APC）的底物，在Arg306与Arg506等位点被裂解后失活[21]–[22]；血浆中大部分TFPIα以与FⅤ B结构域AR2区域结合的形式循环，这种结合不仅为TFPIα提供储存库，也参与其抗凝活性的精细调控，体现出FⅤ作为凝血与抗凝双向调节因子的特点[23]–[25]。

二、TFPIα

TFPI在血浆中以多种亚型形式存在，其中约80％为C端截短形式并多与脂蛋白结合。相比之下，全长的TFPIα虽仅占血浆总TFPI的20％左右，却是参与凝血调控的核心功能形式。TFPIα是一种由276个氨基酸组成、相对分子质量约34×10^3^的分泌型多结构域糖蛋白，含有3个Kunitz抑制结构域（K1、K2、K3）及1个富含碱性残基的C末端尾部[26]–[28]。其中，K1为抑制FⅦa/TF复合物的主要功能域，K2可直接结合并阻断FⅩa活性中心，K3虽无直接蛋白酶抑制活性，却是与辅因子蛋白S（Protein S，PS）相互作用的关键区域。C末端碱性尾段除维持蛋白稳定外，还介导与FⅤ的结合，在凝血调控中具有重要意义[29]–[31]。这种模块化结构赋予TFPIα在多靶点间灵活调控的能力，使其成为外源性凝血途径的核心抑制因子。其抗凝作用依赖于FⅩa驱动的负反馈机制：TFPIα首先通过K2与FⅩa形成复合物，随后该复合物再经K1与TF-FⅦa复合物结合，从而阻断FⅦa对FⅩ的持续激活[29],[32]。这一机制在凝血启动早期即发挥关键抑制作用，防止外源性途径过度放大。

FⅤ与TFPIα的结构域组成如图1所示。

**图1:**
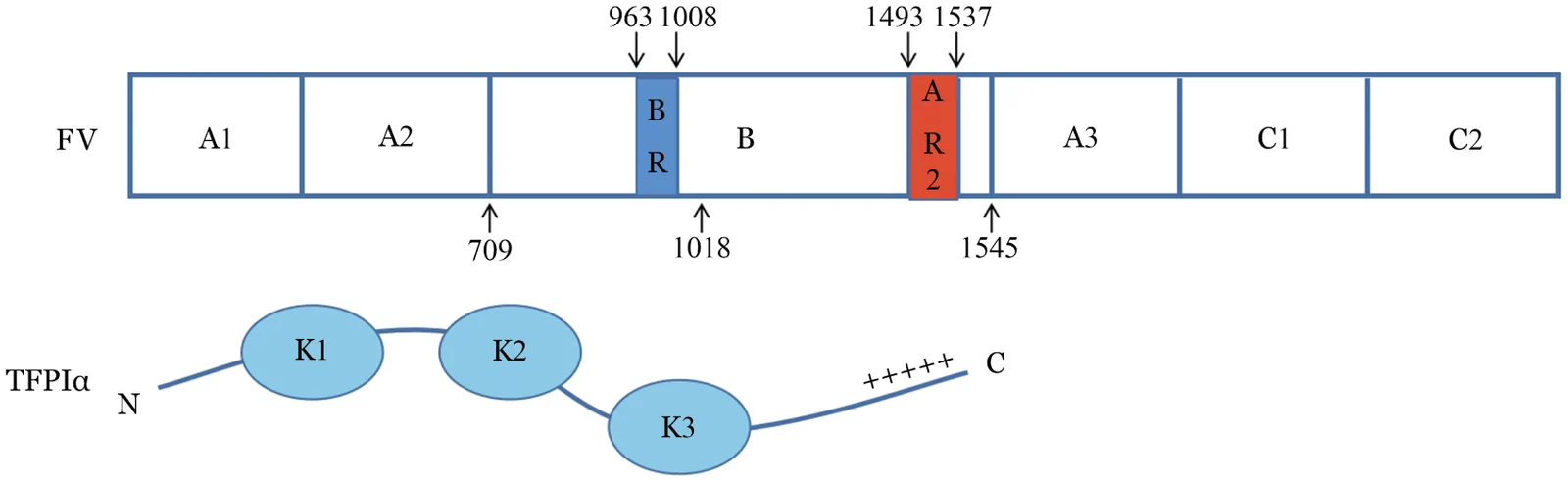
凝血因子Ⅴ（FⅤ）与组织因子途径抑制物α（TFPIα）的结构域组成 **注** FⅤ由A1-A2-B-A3-C1-C2六个结构域构成。B结构域中包含酸性区域（AR2）与碱性区域（BR），在维持FⅤ失活状态及其与TFPIα的相互作用中发挥作用。TFPIα由3个Kunitz结构域（K1、K2、K3）和其C端带正电尾部组成，其中K2域专门调控抑制FⅩa，C端碱性尾则结合FⅤ的AR2区域，以此介导FⅤ-TFPIα复合物的抗凝血机制

三、FⅤ与TFPIα相互作用的分子基础

（一）全长FⅤ中B结构域切割的精密调控

尽管FⅤ的B结构域序列保守性低且高度糖基化，但其内部藏有关键调控位点，包括N端的BR（残基963～1008）与C端的AR2（残基1493～1537）两个区域，二者通过静电相互作用组成自抑制功能模块，使FⅤ维持未激活的前辅因子状态，抑制FⅩa依赖的催化途径激活[2]–[4],[13]。在凝血酶按序切割Arg709和Arg1018后，暴露的AR2特异性结合TFPIα的C端碱性区域，从而暂时阻断Arg1545位点的切割，达成短暂的抗凝作用[6]。Arg1545处被裂解后，AR2片段移除，TFPIα开始解离，FⅤ最终变为FⅤa，并获得促凝活性[2]。这一逐步切割过程准确调节FⅤ从抗凝状态过渡到促凝状态。此外，BR区域部分残基序列与TFPIα的C末端极为相似，说明TFPIα的C末端可能模拟BR的结构界面，从而与AR2暴露区域竞争性或替代性的结合互作，完成对FⅤ活化的精细调控[33]。

（二）FⅤ截短变体（FⅤ-short）的构象激活及其与TFPIα的高亲和力结合

F5基因的一种异常剪接突变可生成FⅤ-short，这是东德克萨斯出血性疾病的分子基础[7]。该突变导致外显子13大段缺失，使B结构域丢失包含BR在内的703个氨基酸而保留AR2及上游疏水斑块。这种结构改变使FⅤ-short无需凝血酶切割即可呈现构象性激活，具备一定的类FⅤa促凝活性[2]，同时暴露的AR2与疏水斑块显著增强其与TFPIα结合，患者血浆中形成大量稳定的FⅤ-short/TFPIα复合物[8]。

研究进一步发现，AR2前端的preAR2区域，尤其是其中PLVIVG（残基1481～1486）疏水斑块对TFPIα发挥抗凝功能至关重要，其为TFPIα/FⅤ-short复合物高效招募PS并产生协同效应所必需[34]–[35]。丙氨酸扫描与AlphaFold分析证实V1483、I1484、G1486等关键残基决定了FⅤ-short/TFPIα/Protein S三元复合物的形成及其FⅩa抑制活性，明确了preAR2区域在协同抗凝机制中的核心作用[36]。

（三）PS对TFPIα抗凝活性的协同增强作用

PS作为维生素K依赖性糖蛋白，以往学界普遍认为其是APC的辅因子[37]–[38]，但近年来发现PS还是TFPIα的核心辅助因素，在FⅤ-TFPIα复合物的形成与功能放大中发挥关键作用[39]–[41]。PS大幅增强TFPIα与膜表面及FⅩa的相互作用，抑制作用进一步加大[42]–[43]，在FⅤ-short存在时，这种协同更为突出，三者形成稳定的分子复合物，对FⅩa的抑制作用显著提升（图2）。AlphaFold结构预测可知，FⅤ-short B结构域的疏水斑块可能会与PS两处疏水区域（残基268～276和422～426）直接相互作用，并联合TFPIα C端片段增强复合物稳定性，大幅降低凝血酶产生量[23],[36],[44]。因此，PS除了参与APC途径调控，还是FⅤ-TFPIα凝血调控网络的关键成分，这表明FⅤ-short/TFPIα/PS复合物可能成为潜在的抗凝干预有效靶点。

**图2:**
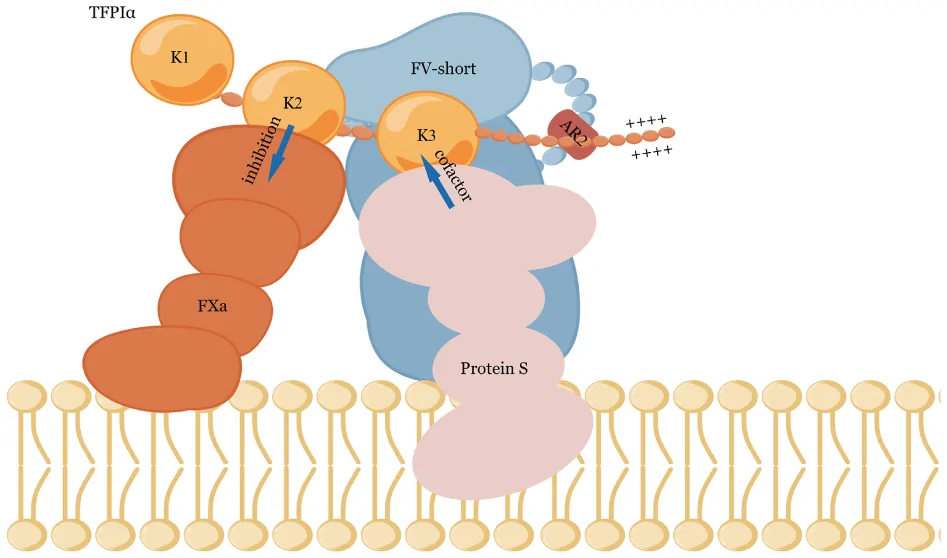
FⅤ-short/TFPIα/PS三元复合物在磷脂膜上发挥抗凝作用 **注** 在磷脂膜表面，凝血因子Ⅴ截短变体（FⅤ-short）暴露出的酸性区域（AR2）与组织因子途径抑制物α（TFPIα）的C端碱性尾结合，蛋白S（PS）通过与TFPIα的K3结构域相互作用进一步稳定该复合物，并促进其定位于膜表面。在此构象下，TFPIα的K2结构域可直接抑制FⅩa的活性，从而阻断凝血级联的放大反应（使用BioGDP.com绘制[45]）

四、冷冻电镜解析下的FⅤ构象特征

（一）全长FⅤ与FⅤa的整体结构特征

近年来，冷冻电镜技术的发展为解析大型蛋白质复合物高分辨率结构实现了突破。Ruben等[10]首次报道了人源FⅤ与FⅤa的冷冻电镜结构，显示C1和C2结构域共同构成磷脂膜结合平台，支撑着A1和A3结构域，A2结构域处于最顶端。FⅤ与FⅤa整体构象大致相似，但FⅤa中A2结构域C末端的柔性增强明显，这个区域正是凝血酶原结合的核心位点。同时，FⅤa的APC裂解位点R306和R506的可及性由FⅤ的25％升至60％，为其更易被灭活提供了结构学角度的依据。

（二）FⅤ-short的有序结构及其固有活性机制

尽管全长FⅤ中B结构域的高度无序性阻碍了其结构解析，但剪接变体FⅤ-short在冷冻电镜下却呈现出稳定而清晰的构象。该变体的B结构域缺失703个氨基酸（残基756～1458），仅保留133个残基，形成一条横跨A1、A2、A3的残余B链，悬于C1、C2之上但与三者形成多点相互作用。A2结构域发生显著构象重排，使关键残基Tyr696与Arg701的侧链朝向趋于FⅤa型，同时暴露酸性片段^658^PDDDEDSYEIFEPP⁶⁷¹，其中⁶⁵⁹DDDED⁶⁶³簇可调控凝血酶原裂解顺序与速率，增强凝血酶生成[46]。因此，FⅤ-short即便未经凝血酶切割，A2域也能形成一个类似于FⅤa的、易于与FⅩa结合的平台，从结构上解释了其固有的促凝活性[9]。

此外，FⅤ-short中PLⅤIVG疏水斑块与AR2区共同组成一个结构稳定、表面暴露的酸性模块，与因构象重排而暴露的A2结构域酸性片段相邻，形成连续负电表面以招募带碱性尾的TFPIα。全长FⅤ中该位点由BR片段自抑制而被遮蔽，从而维持非活化构象，而在FⅤ-short中则因BR缺失而暴露，可与TFPIα结合（图3）。该结合界面还在空间上靠近APC裂解位点R506与凝血酶切割位点R1545，结合TFPIα后可物理遮蔽二者，阻碍相应蛋白酶的切割。因此，TFPIα通过其C端尾锚定于FⅤ-short表面后，其Kunitz结构域便能有效抑制FⅩa，最终阻断FⅤ-short参与形成的凝血酶原酶复合物活性[9]。

**图3:**
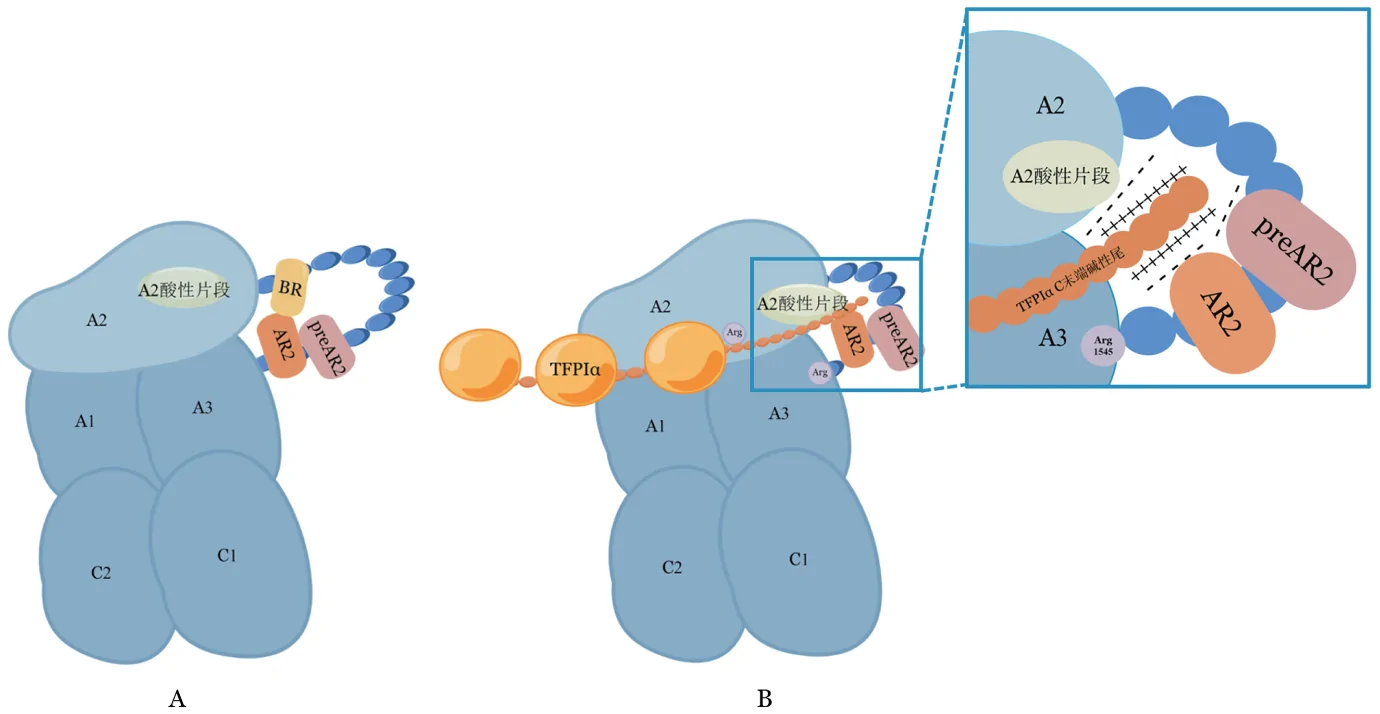
凝血因子Ⅴ截短变体（FⅤ-short）与组织因子途径抑制物α（TFPIα）C末端尾部结合的结构示意图 **A** 全长FⅤ；**B** FⅤ-short **注** 在FⅤ-short缩短的B结构域上，暴露的AR2和疏水斑块preAR2与A2结构域上因构象重排而暴露的酸性片段（^658^PDDDEDSYEIFEPP^671^），共同形成一个扩展的负电表面，TFPIα的C末端碱性尾部与此结合表位结合。图中还标注了R506与R1545位点，它们在TFPIα结合后被遮蔽（使用BioGDP.com绘制[45]）

（三）全长FⅤ中AR2结构的保守性及关键残基的探索

冷冻电镜解析揭示了FⅤ-short B结构域中AR2区域的真实构架，但其是否为剪接特有结构仍存疑。近期基于改进算法的全长FⅤ冷冻电镜重解析显示，在残基1507～1545区域存在与FⅤ-short中AR2高度相似的构象，提示AR2在全长FⅤ中同样保守存在，并通过与BR的分子内相互作用维持FⅤ的非活化状态[47]。

在此基础上，对AR2关键相互作用残基的探索进一步深入。早期研究依赖圆二色性与分子对接等预测手段，提出若干可能通过盐桥或氢键介导与BR或TFPIα结合的残基[48]。然而，冷冻电镜实测结构表明部分预测残基因空间朝向或溶剂暴露状态限制，缺乏直接结合能力[9]。这些结果提示，仅依赖模型推测不足以揭示真实的分子界面，需结合高分辨率结构与定点突变等实验手段，才能更准确地阐明FⅤ-TFPIα相互作用的分子基础。

五、FⅤ-TFPIα相互作用的病理失调

如前所述，TFPIα仅占血浆总TFPI的20％左右，在正常血浆中可与FⅤ相互作用。Duckers等[49]通过免疫耗竭实验发现，在生理钙离子浓度下去除血浆中的FⅤ后，TFPIα水平下降约80％，提示血浆中大部分TFPIα以与FⅤ结合的形式存在。这种相互作用有助于TFPIα在凝血启动部位发挥对FⅩa的抑制作用，是维持凝血稳态的重要机制。由于这种相互作用的生理功能至关重要，当其因基因突变而发生紊乱时，就可能会造成出血或血栓的倾向。

（一）FⅤ-TFPIα相互作用增强导致的出血倾向

1. F5-east Texas：东德克萨斯出血性疾病由F5基因c.2350A>G剪接位点突变引起，产生FⅤ-short变体。该变体能与TFPIα形成大量稳定复合物，并在PS协同下成为高效的FⅩa抑制因子，过度抑制凝血过程中的关键蛋白酶FⅩa。尽管患者凝血因子水平正常，但凝血酶生成显著受损，表现为轻至中度终身性出血倾向，如鼻出血、月经过多及术后出血[3],[50]。

2. F5-Amsterdam：F5-Amsterdam源于F5基因外显子13上的c.2588C>G突变，产生一个新的供体剪接位点，导致mRNA异常剪接并生成缺失623个氨基酸的缩短型FⅤ变体。与F5-east Texas类似，该变体缺失B结构域包含碱性区的大片段，对TFPI具有高亲和力，造成循环TFPI水平显著升高，凝血酶生成受阻并引起家族性出血倾向[51]。

3. F5-Atlanta：F5-Atlanta突变（c.2413_3244del，p.Thr805Serfs*13）通过另一机制产生FⅤ-short。该突变导致外显子13中位于FⅤ-east Texas突变的3′端缺失832个碱基对，推测去除了一个或多个剪接沉默子，解除了对FⅤ-short特异性剪接的抑制，使得等位基因大多生成FⅤ-short转录本，患者血浆中FⅤ-short显著增多[52]–[53]，出血情况比东德克萨斯出血性疾病更严重。

（二）FⅤ缺乏对TFPIα水平及功能的影响

除上述可增强TFPIα结合的罕见变体外，在同样由F5基因突变引起的、以出血倾向为特征的遗传性FⅤ缺乏症中，FⅤ水平的降低可对其与TFPIα相关的相互作用产生影响。循环FⅤ的显著缺乏不仅损害了促凝功能，还导致TFPIα失去了其主要的血浆结合蛋白，可能进而影响TFPIα稳定性和清除。这种继发性的TFPIα水平降低，反而减弱了对凝血级联的抑制作用，使有限的FⅤ仍能相对有效地参与凝血酶生成，从而部分代偿了原发性的促凝缺陷，这也许是部分患者出血症状轻于预期的原因[49]。由此可见，FⅤ作为TFPIα的主要结合蛋白，二者之间的物理与功能联系在维持凝血与抗凝平衡中发挥着重要调控作用。

六、研究展望与治疗前景

目前，针对FⅤ相关出血或血栓性疾病的治疗仍主要依赖非特异性方案，如血浆输注、抗纤溶药物或常规抗凝剂，尚未直接靶向FⅤ-TFPIα相互作用这一关键环节[54]–[58]。随着其分子机制的深入揭示，基于FⅤ-TFPIα轴的精准治疗显示出巨大潜力。例如，可设计模拟TFPIα C端尾部的小分子以增强抗凝，或开发竞争性抑制剂阻断TFPIα结合以提高FⅤ促凝活性。尽管这些策略仍处探索阶段，但已指明了从传统替代治疗向靶向精准调控的转变。近年来靶向TFPI的单克隆抗体已在血友病治疗中开展临床研究并显示出良好前景，通过阻断TFPI的抗凝作用重建凝血平衡，进一步支持了TFPI作为重要调控靶点的临床转化潜力[59]。未来研究仍面临挑战：目前缺乏高分辨率的FⅤ-TFPIα复合物结构，限制了结合界面解析；而对罕见变体（如F5-east Texas）的认识亦局限于个案报道，缺乏大样本流行病学与系统功能学数据。后续工作应结合多组学分析、结构生物学、基因编辑动物模型及临床研究，系统阐明FⅤ-TFPIα相互作用的分子基础与体内功能，为出血与血栓性疾病的精准防治提供新思路。
